# Revision surgery for periprosthetic fracture of distal femur after endoprosthetic replacement of knee joint following resection of osteosarcoma

**DOI:** 10.3389/fonc.2024.1328703

**Published:** 2024-02-12

**Authors:** Qing-lin Jin, Hao-bin Su, Shao-hua Du, Chang-he Hou, Ming Lu, Shuang-wu Dai, Zi-xiong Lei, Wei Chen, Hao-miao Li

**Affiliations:** Department of Musculoskeletal Oncology, Center for Orthopaedic Surgery, The Third Affiliated Hospital of Southern Medical University, Guangzhou, China

**Keywords:** osteosarcoma, periprosthetic fracture, distal femur, limb salvage, revision surgery

## Abstract

**Purpose:**

Periprosthetic fracture (PPF) is one of the severe complications in patients with osteosarcoma and carries the risk of limb loss. This study describes the characteristics, treatment strategies, and outcomes of this complication.

**Methods:**

Patients were consecutively included who were treated at our institution between 2016 and 2020 with a PPF of distal femur. The treatment strategies included two types: 1) open reduction and internal fixation with plates and screws and 2) replacement with long-stem endoprosthesis and reinforcement with wire rope if necessary.

**Results:**

A total of 11 patients (mean age 12.2 years (9–14)) were included, and the mean follow-up period was 36.5 (21–54) months. Most fractures were caused by direct or indirect trauma (n = 8), and others (n = 3) underwent PPF without obvious cause. The first type of treatment was performed on four patients, and the second type was performed on seven patients. The mean Musculoskeletal Tumor Society (MSTS) score was 20 (17–23). All patients recovered from the complication, and limb preservation could be achieved.

**Conclusion:**

PPF is a big challenge for musculoskeletal oncologists, particularly in younger patients. Additionally, PPF poses a challenge for orthopedic surgeons, as limb preservation should be an important goal. Hence, internal fixation with plates and endoprosthetic replacement are optional treatment strategies based on fracture type and patient needs.

## Introduction

Osteosarcoma (OS) is the most common primary malignancy of bone in children and adolescents, while primary bone neoplasms account for merely less than 0.2% of all cancers (1). Since the 1980s, there has been a consensus in osteosarcoma treatment that a combination of surgery and intensive, multi-agent chemotherapy dramatically improves osteosarcoma patients’ prognosis and maintains a high survival rate and long-term disease-free survival (2–4).

Various techniques have been reported for reconstruction after malignant tumor resection around the knee in children and adolescents (5, 6). However, revision of endoprosthetic reconstructions occurs frequently because of infection and mechanical complications (5, 7–10). In the context of endoprosthetic reconstruction for distal femoral tumors, the respective proportions of complications according to systematic classification are soft-tissue failure (4.6%), aseptic loosening (24.9%), structural failure (23.0%), infection (30.3%), and tumor recurrence (17.2%) (11). With the survival of osteosarcoma patients being improved, combined with most patients being young and active and with poor bone quality, the rate of periprosthetic fracture (PPF) is increasing among mechanical complications. PPF is one of the most common complications experienced by patients with malignant tumors around the knee joint (12, 13). Furthermore, in a study focusing on adolescent patients with bone tumors under the age of 18, the rates of periprosthetic fractures at 5 and 8 years were higher than those of infection and aseptic loosening (13).

The risk factors for PPF can be broadly categorized into three main groups. Patient-specific factors include diabetes, osteoporosis, age, or undergoing certain specific treatments. Implant-related factors encompass the design of implant components, such as the presence of collars. Lastly, surgical factors will involve considerations like notching at the anterior femoral cortex, surgical volume, and intraoperative mishandling. However, there is still controversy surrounding many of these factors (14, 15). Currently, various classification systems can be adopted for the treatment of periprosthetic fractures, depending on different focal points, including conservative treatment, open reduction internal fixation, and revision reconstruction. However, most patients with PPF need surgical treatment finally. At present, the Unified Classification System (UCS) classification system serves as the mainstream basis for selecting treatment approaches for periprosthetic fractures (16, 17). However, there are no widely accepted guidelines or consensus to directly treat PPF patients after limb salvage surgery caused by OS of the knee joint yet.

To our knowledge, very rare reports or recommendations have demonstrated treatment strategies for PPF with patients who experienced osteosarcoma around the knee joint (18–20). In this study, we aimed to produce different types of PPF and describe 13 cases with successful treatment of PPF with limb-salvaging strategies.

## Patients and methods

### Study design

Patients were consecutively included who were treated at our institution between 2016 and 2020 with a PPF of distal femur. A total of 11 patients have been included who 1) were treated at our institution with the diagnosis of PPF, 2) were treated between 2016 and 2020, 3) have undertaken prosthetic reconstruction surgery for osteosarcoma of the knee joint, 4) were younger than 14 years, and 5) were with complete clinical, radiological, and pathological data. The exclusion criteria included the following: 1) evidence of peri-prosthetic infection, 2) evidence of local tumor recurrence, 3) patients who had unfinished treatment of initial osteosarcoma, 4) patients who received specific treatment in other case–control studies, and 5) patients with a Karnofsky performance score <70. As for the types of PPF, the UCS was used for the classification (17). Additionally, PPF was classified according to the location and stability of the prosthesis: type I, around the tip of the prosthesis stem, or the prosthesis is unstable; and type II, away from the tip of the prosthesis, and the prosthesis is still well fixed, too. The follow-up interval for primary osteosarcoma followed National Comprehensive Cancer Network (NCCN) guidelines (1). The study was approved by the institutional review board (IRB) of our institutions and was conducted in accordance with the Declaration of Helsinki.

### Treatment procedures

#### Internal fixation with plates and screws

X-ray positioning was used during surgery to determine the surgical approach. The fractured part was exposed, and the surrounding hematoma, osteocytes, and tissues in the medullary cavity were cleaned. Meanwhile, rapid pathological examination of intramedullary tissues was performed to eliminate the recurrence of the tumor. Then, the fracture was reduced by traction and fixed using locking plates (shaped according to bone shape) and screws. Locking-nail channels blocked by the prosthesis stem were fixed with a single cortical screw and wire rope. After fixation and defining reduction with X-ray, allogeneic/autologous bone was implanted around the fracture line of the femur.

#### Replacement of endoprosthesis

The fractured part was exposed, and the prosthesis of the knee joint was displaced. The affected part of the prosthesis was removed from the medullary cavity, and the stability of the unaffected part of the prosthesis was checked. The hyperplastic granulation tissue and bone cement in the medullary cavity were removed and flushed for 3 cycles. The antibiotic bone cement was injected, and a new custom-made prosthesis with a longer stem was installed. The position of the prosthesis was confirmed by an X-ray, and the fractured part was reinforced with wire rope if necessary.

### Follow-up

The patients were followed every 3 months for the first and second years postoperatively and then every 6 months for three more years. After that, follow-up was performed once a year. Each follow-up visit included history taking, physical examination, and radiological examination, such as X-ray and CT scan for mechanical complication and tumor recurrence. PET-CT was used to assess the tumor’s metastatic status. The postoperative functional assessment was performed via the Musculoskeletal Tumor Society (MSTS) Functional Scoring System (21).

## Results

### Patients’ characteristics

Overall, 11 patients (11/313, 3.5%) with PPF were included in this study. The mean follow-up time was 36.5 (21–54) months. According to the UCS classification (17), these patients were classified as type B1 in five (45.5%) and type B2 in six (54.5%). According to the classification of our institution, seven (63.6%) patients were type I, and four patients (36.4%) were type II. Eight (72.7%) fractures were caused by direct or indirect trauma, while three (27.3%) fractures underwent PPF without obvious cause. Four (36.4%) patients with type II PPF underwent fixation with plates and screws, and seven (63.6%) patients with type I PPF underwent replacement of endoprosthesis with further reinforcement (**Table 1**). The mean MSTS score was 20 (17–23). Until the last follow-up, none experienced any complications such as infection, delayed wound healing, re-fracture, or non-union. No re-revision was necessary in all cases.

**Table 1:** Clinical characteristics of the patients.

Patient	Age (years)	Gender	IT (month)	Cause	UCSC	Type	Treatment	Follow-up (month)	MSTS	Complication
1	14	F	8	Fall	B2	I	PR	54	21	No
2	10	M	10	Fall	B1	II	P&S	51	17	No
3	9	M	44	WOC	B1	I	PR	27	19	No
4	9	F	66	WOC	B2	I	PR	49	20	No
5	12	F	14	Fall	B2	I	PR	33	22	No
6	14	M	12	Sprain	B1	II	P&S	34	21	No
7	13	M	22	Fall	B1	II	P&S	32	20	No
8	14	F	23	Fall	B2	I	PR	22	22	No
9	14	F	37	WOC	B1	I	PR	45	23	No
10	11	M	55	Fall	B2	II	P&S	33	19	No
11	14	F	34	Fall	B2	I	PR	21	18	No

IT, interval time between periprosthetic fracture and initial limb-salvage surgery; F, female; M, male; WOC, without obvious cause; UCSC, Unified Classification System classification; P&S, internal fixation with plates and screws; PR, prosthesis replacement; MSTS, Musculoskeletal Tumor Society Functional Scoring System.

### Special cases

#### Case 1

**Figure 1:**
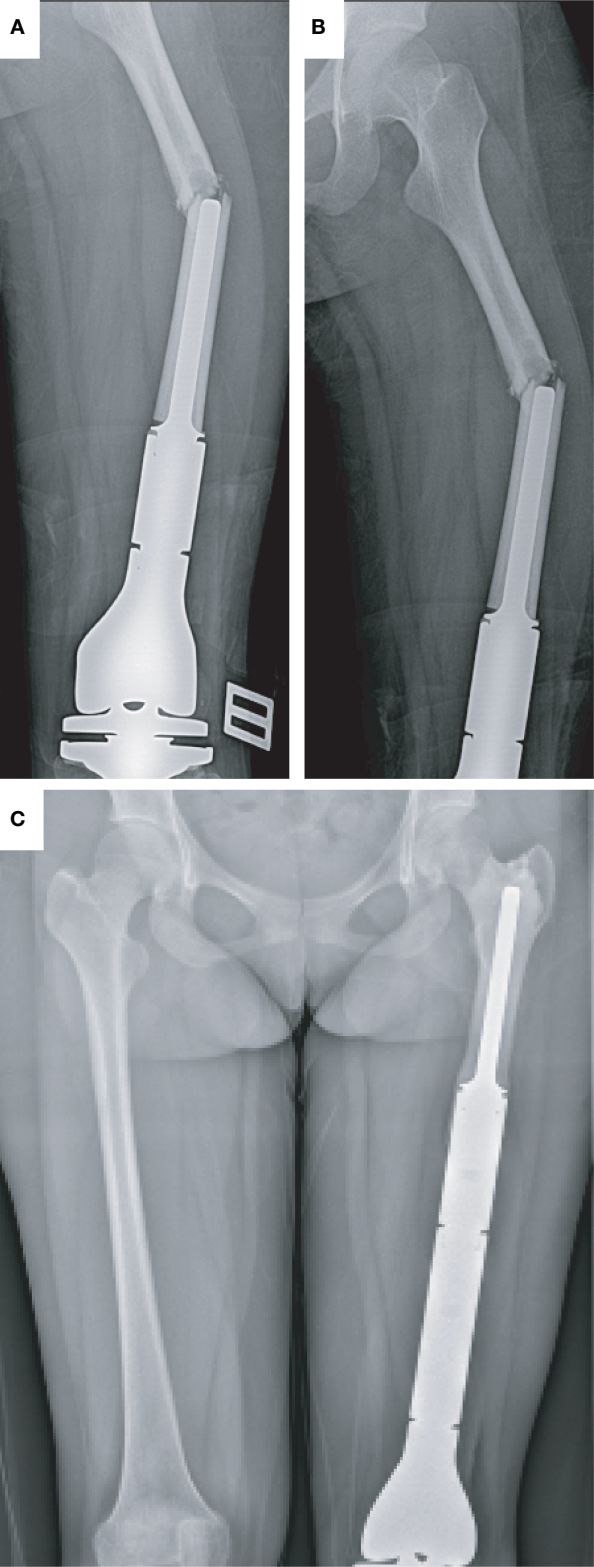
Type I periprosthetic fracture and corresponding surgical strategy (open reduction and replacement of endoprosthesis). **(A, B)** Preoperative X-ray image of the left femoral fracture and **(C)** postoperative X-ray image of the bilateral femur.

A 14-year-old girl suffered from a PPF at the left femur after falling. This patient complained of swelling and pain in the left leg. From the X-ray image, PPF in the middle part of the left femur with partial displacement (Type I) was observed (**Figures 1A, B**). Revision surgery with the replacement of a custom-made prosthesis stem was performed (**Figure 1C**). According to the last follow-up data, the patient was alive, and no postoperative complication happened.

#### Case 2

**Figure 2:**
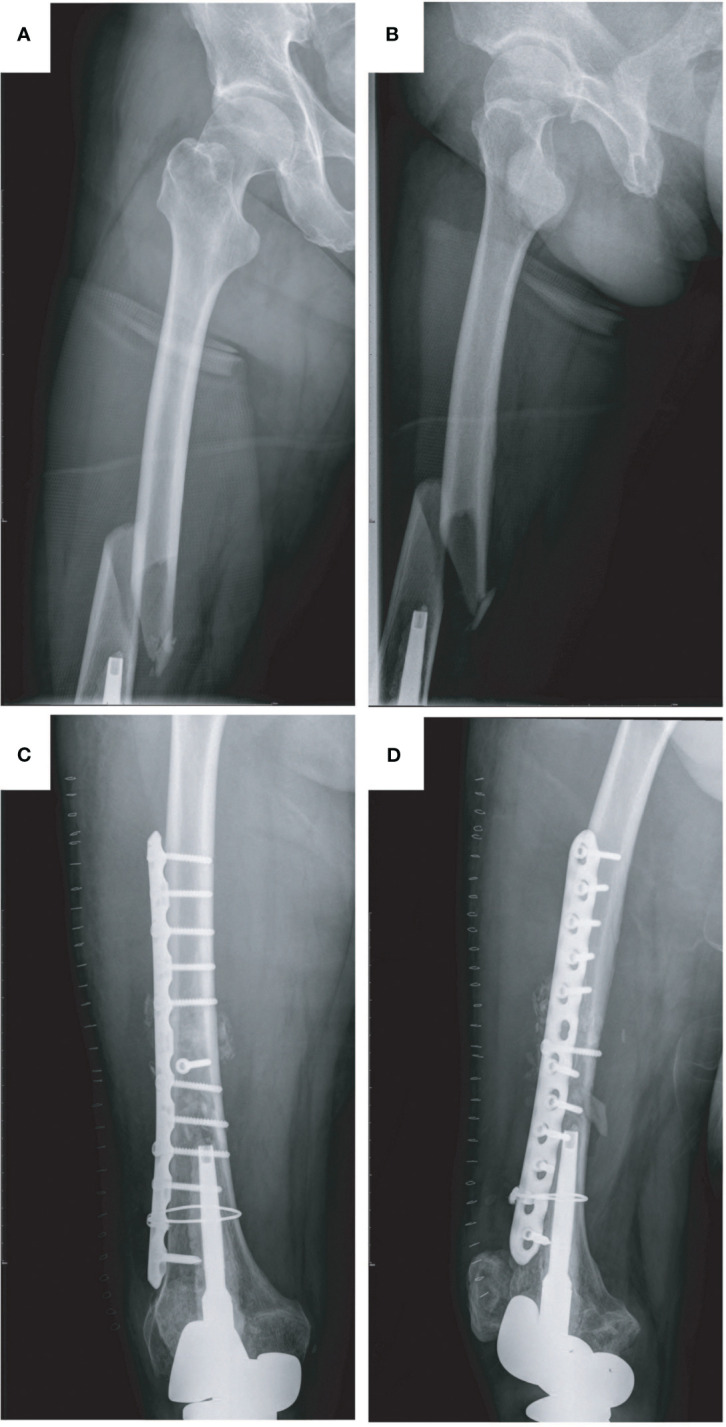
Type II periprosthetic fracture and corresponding surgical strategy (open reduction and internal fixation with plates and screws, reinforcement with wire rope). **(A, B)** Preoperative X-ray image of the right femoral fracture and **(C, D)** postoperative X-ray image of the right femur.

A 14-year-old boy with right total knee prosthetic reconstruction after resection of tibial osteosarcoma had a sprain and suffered from a prosthetic femoral fracture at the middle part of the right femur (Type II) (**Figures 2A, B**). At the first visit, the affected limb was swelling and deformed with excruciating pain in the right thigh. After taking a careful physical examination and X-ray imaging of the affected limb and confirming the fracture site, type, and condition of the bone cortex, revision surgery with plate osteosynthesis was performed. At the last follow-up, no further complication happened, and the patient was fully mobilized (**Figures 2C, D**).

## Discussion

Artificial prostheses are frequently used to reconstruct bone defects caused by resection of a bone malignancy from the distal femur. However, substantial mechanistic complication rates should be considered because of the severe situation, most notably for aseptic loosening and periprosthetic fracture (9, 20, 22–24). Henderson et al. reported that periprosthetic fracture was the most frequent type of mechanical failure with a rate of 17% followed by aseptic loosening (24). D. Andreou reported that structural complication including PPF and implant fracture is the most common complication experienced by patients with malignant tumors around the knee joint (12). From the database of our institution, nearly 17.7% (11/62) of patients experienced PPF among all patients of mechanical failure after prosthesis reconstruction. Notably, we should focus on PPF since it is one of the most common complications.

For traditional non-tumoral implants, the mean interval between implantation and PPF was 4.4 to 6.4 years (25–29). Periprosthetic fractures are often directly caused by trauma, and the risk factors for their occurrence are closely related to the patient’s own conditions, such as diabetes, osteoporosis, or severe bone loss, all of which contribute to decreased bone quality. The differences in components of the initial reconstruction implant, the width ratio of bone to implant, and the elastic modulus disparity between components and the recipient bone bed are also influencing factors (14, 15). However, the choice of fixation methods and hinge mechanisms for implants has no statistically significant impact on the occurrence of periprosthetic fractures following initial distal femoral reconstruction (30).

For patients who underwent total knee reconstruction after malignant tumor resection, the mean interval between first implantation and revision caused by PPF was significantly shorter than in those patients with non-tumoral disease (18, 19, 23). Andreou et al. (12) reported that the mean time to first mechanical complication for patients with bone tumors was 16 months. In this study, the mean age at the time of fracture was 12.2 years (range, 9–14), and the mean time from revision to implantation was 31 months (range, 6–65). This difference is owing to the primary diagnosis that led to the reconstruction. PPFs around tumor endoprosthesis are very different from those of standard implants. In this study, PPFs were classified as B1 in five (45.5%) cases and B2 in six (54.5%) cases according to UCS classification. Spina et al. (27), in a series of 61 periprosthetic femoral fractures, reported that nearly 80% of patients were classified as type B fractures, while only 2% were classified as type A. For a series of cases with tumor endoprosthesis, Andreou et al. (12) showed that over 65% of patients with PPF were within UCS type C fracture, and only 17% of patients were classified in type B fracture. There is not enough evidence to prove any difference between tumors and conventional prostheses. It is clear that trauma is a direct cause of PPF. In this study, which focuses on adolescent osteosarcoma patients, standardized chemotherapy during the preoperative and postoperative periods in the presence of immature bone can lead to decreased bone healing and poorer bone mass. In addition, younger patients are at increased risk of fracture due to being more physically active (13, 31). At the same time, the mismatch between the physiological curvature of the bone and the lateral position of the tumor prosthesis, the angle of curvature, and the actual length of these endoprostheses concentrate on stresses topically, leading to progressive destruction of the femoral cortex. Moreover, the shorter length of the remaining stable bone during the initial reconstruction to achieve complete tumor resection, as well as the soft-tissue stripping and diminished protection of the normal adjacent musculoskeletal tissues, significantly elevates the chances of fracture (32, 33).

PPF is a big challenge for patients and oncologists. The main goals of the treatment are limb preservation, preservation of function, and reduction of the re-revision rate. Most importantly, PPF accounted for the vast majority of the second revision followed by infection (10, 12, 13). The presented 11 cases demonstrated that even in such complex situations, successful fracture treatment and reducing the rate of complication were possible and critical for patients, especially young adolescent patients.

In this study, we classified the PPF into two types: type I, around the tip of the prosthesis stem, or the prosthesis is unstable (unable to fix the bone and prosthesis firmly via plates and screws); type II, away from the tip of the prosthesis, and the prosthesis is still well fixed (there was enough space for fixing the fractured bone firmly with plates and screws). Meanwhile, we demonstrated two ways of revision surgery according to the types of PPF: 1) P&S (open reduction and internal fixation with plates and screws) and 2) replacement of prosthesis with longer stem for different types of PPF. Revision surgery is recommended for the anatomical reduction of the fracture and the stability of the prosthesis, which could lead to early limb movement and the recovery of affected limbs. For patients with type I PPF, we recommended open reduction and replacement of the prosthesis with a longer stem. For several patients, we found that the prosthesis stem was unstable even though the fractured part was away from the tip of the stem. Therefore, we recommended prosthesis replacement instead of fixation with plates and screws. For patients with type II PPF, we recommended open reduction and internal fixation with plates. On the one hand, the residual bone was closely connected to the prosthesis stem and stable. On the other hand, there is enough bone mass and space around the fracture site for fixation with plates and screws. Hence, fixation with plates and screws was recommended considering the economic condition of patients. Up to now, those patients recovered successfully without any other complications from the first revision surgery. Fortunately, the mean MSTS score of this study was 20 points, and all patients were satisfied with the therapeutic effect of our revision surgery.

One of the main limitations is the low number of cases. In this study, we proposed two different types of surgical procedures to treat PPF in different types and achieve the goal of individualized treatment. Another limitation is that the time of follow-up of several cases is too short, and further follow-up was needed. Therefore, more clinical practice is needed to validate our proposed treatment strategy for different types of PPF.

## Conclusion

PPF is a significant concern for musculoskeletal oncologists, particularly in younger patients. Additionally, PPF poses a challenge for orthopedic surgeons, as limb preservation should be an important goal. The difficulty lies in different types and severity and needs different means to work out. Minimizing complications and improving stability are the key to success. We demonstrate two types of procedures. The preservation of the extremity should always be the primary goal.

## Data availability statement

The raw data supporting the conclusions of this article will be made available by the authors, without undue reservation.

## Ethics statement

The studies involving humans were approved by The Third Affiliated Hospital of Southern Medical University. The studies were conducted in accordance with the local legislation and institutional requirements. Written informed consent for participation in this study was provided by the participants’ legal guardians/next of kin. Written informed consent was obtained from the minor(s)’ legal guardian/next of kin for the publication of any potentially identifiable images or data included in this article.

## Author contributions

Q-LJ: Investigation, Methodology, Writing – original draft, Writing – review & editing. H-BS: Formal analysis, Investigation, Writing – original draft, Writing – review & editing. S-HD: Investigation. C-HH: Data curation. ML: Data curation. S-WD: Writing – review & editing. Z-XL: Writing – review & editing. WC: Project administration, Writing – review & editing. H-ML: Project administration, Supervision, Writing – review & editing.
